# Stimuli-responsive DNA-based hydrogels for biosensing applications

**DOI:** 10.1186/s12951-022-01242-x

**Published:** 2022-01-21

**Authors:** Mengmeng Chen, Yu Wang, Jingyang Zhang, Yuan Peng, Shuang Li, Dianpeng Han, Shuyue Ren, Kang Qin, Sen Li, Zhixian Gao

**Affiliations:** Tianjin Key Laboratory of Risk Assessment and Control Technology for Environment and Food Safety, Tianjin Institute of Environmental and Operational Medicine, Tianjin, 300050 People’s Republic of China

**Keywords:** DNA hydrogel, Stimuli-responsiveness, Biosensing

## Abstract

The base sequences of DNA are endowed with the rich structural and functional information and are available for the precise construction of the 2D and 3D macro products. The hydrogels formed by DNA are biocompatible, stable, tunable and biologically versatile, thus, these have a wide range of promising applications in bioanalysis and biomedicine. In particular, the stimuli-responsive DNA hydrogels (smart DNA hydrogels), which exhibit a reversible and switchable hydrogel to sol transition under different triggers, have emerged as smart materials for sensing. Thus far, the combination of the stimuli-responsive DNA hydrogels and multiple sensing platforms is considered as biocompatible and is useful as the flexible recognition components. A review of the stimuli-responsive DNA hydrogels and their biosensing applications has been presented in this study. The synthesis methods to prepare the DNA hydrogels have been introduced. Subsequently, the current status of the stimuli-responsive DNA hydrogels in biosensing has been described. The analytical mechanisms are further elaborated by the combination of the stimuli-responsive DNA hydrogels with the optical, electrochemical, point-of-care testing (POCT) and other detection platforms. In addition, the prospects of the application of the stimuli-responsive DNA hydrogels in biosensing are presented.

**Graphical abstract:**

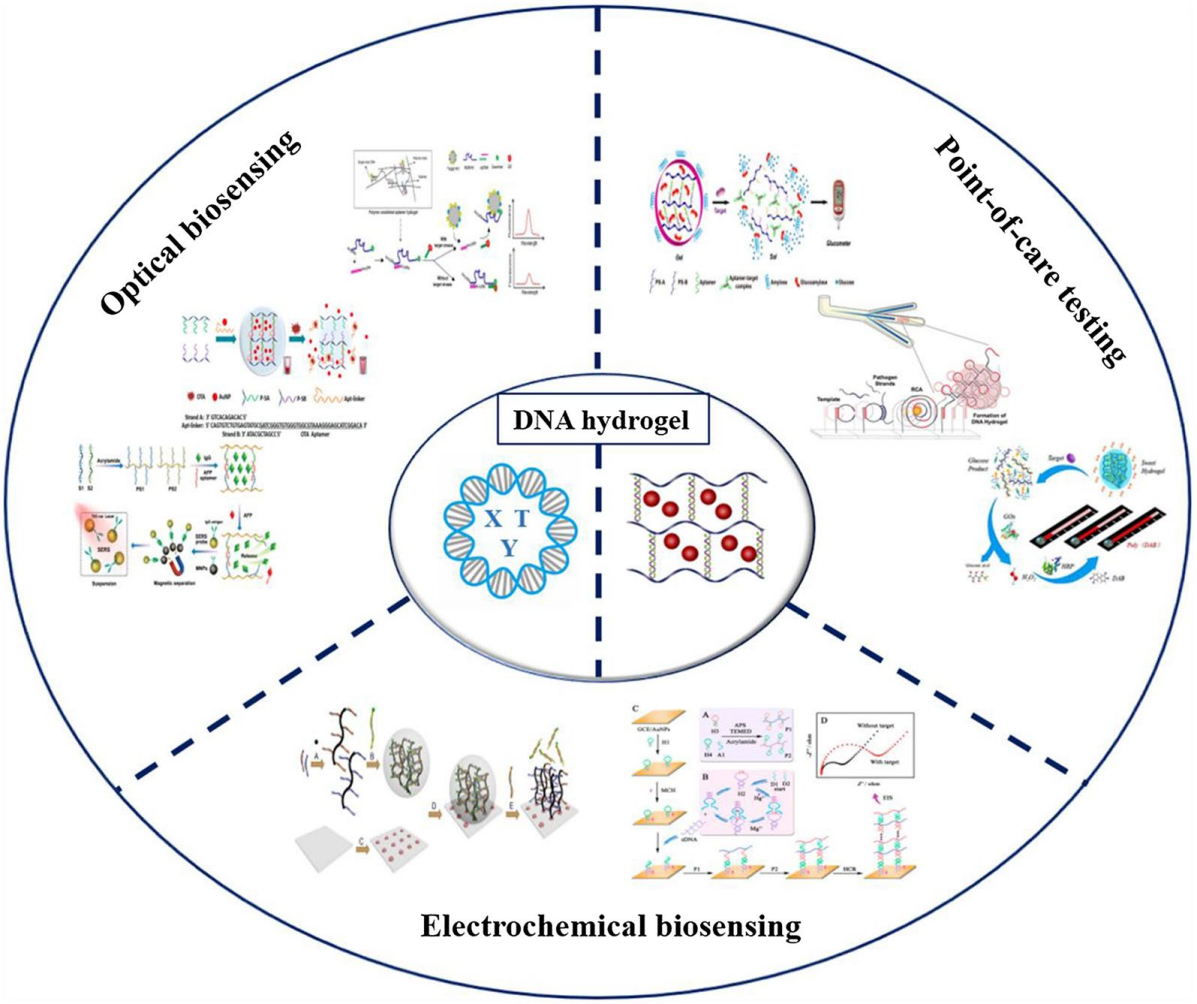

## Introduction

DNA has existed in nature for billions of years, and the discovery of the double helix structure of DNA by Watson and Crick in 1953 ushered in the possibilities for understanding and application of DNA at the molecular level [1]. In recent years, the advances in the DNA synthesis technology have facilitated its use as a functional material in chemistry [2], physics [3], computer science [4, 5] and medicine [6]. Due to its stability, flexibility, precise programmability, ease of synthesis and modification, etc., DNA can be accurately manipulated to generate various DNA building blocks with unique geometric structures, thus, resulting in the highly predictable and structured DNA networks [7–11]. In addition, the functional DNA structures can also undergo the conformational transformations in response to the external stimuli, such as the assembly of the guanine (G)-rich strands into G-quadruplexes on K^+^ stimulation, which further promotes them as the functional building blocks for the construction of the responsive materials [12].

The hydrogels prepared by chemical bonding among the DNA molecules or physical entanglement among the DNA strands are the biocompatible polymeric network materials which can absorb a large amount of water, thus, causing them to swell to hundreds of times the dry weight of the gel [13]. Of significant interest are the stimulus-responsive DNA hydrogels or smart DNA hydrogels. In the presence of the external triggers, such as pH [14–19], light [20], temperature [21–23], ionic strength [24–26], electric field strength [27], ultrasonic radiation [28] and magnetic stimulation [29, 30], the responsive DNA hydrogels are stimulated to undergo changes in crosslinking density at the microscopic level. This ensues the reversible and switchable hydrogel-to-sol or hydrogel-to-solid transitions at the macroscopic scale, which makes the hydrogels highly promising for drug delivery [31–36], controlled release [37–41], tissue engineering [42–44] and sensing applications [45–47]. Especially in the field of sensing, the DNA hydrogels combined with the diverse sensing platforms react with the metal ions [48, 49], nucleic acids [50–54], proteins [55, 56] and carcinogens [57], thereby converting them as changes in DNA hydrogel material properties, allowing the sensitive and specific target detection [58, 59]. Therefore, the DNA hydrogels are the functional platforms to detect a wide range of stimuli under different conditions.

Based on their components, DNA hydrogels can be divided into two groups, hybridized and pure DNA hydrogels. The hybridized hydrogels are formed by cross-linking the DNA molecules grafted on the hydrophilic polymer chains as interaction nodes, which was originally proposed by Nagahara and Matsuda [60]. The authors grafted single-stranded DNA (ssDNA) onto the polyacrylamide chains and subsequently formed the DNA hydrogels based on the base complementary pairing cross-linking between ATs. Using the similar strategy, the DNA-polyacrylamide chains have been used to construct various functional hydrogels, which have been applied in many fields, especially in the stimulus-responsive systems. In contrast, the pure DNA hydrogels are entirely made of the DNA molecules. These are generally assembled by enzymatic ligation, polymerization, hybridization, and specific binding of the DNA motifs as building blocks. In comparison with the hybridized hydrogels, the pure DNA hydrogels are more biocompatible, biodegradable, and easily moldable into the desired shapes and sizes. The preparation of the DNA hydrogels via nucleic acid amplification leads to the cost-effective synthesis and further expands the practical applications of the pure DNA hydrogels.

In this review, the different approaches to construct the DNA hydrogels have been presented. Subsequently, using selected examples, the specific applications and recent advances of the stimuli-responsive DNA hydrogels in the field of biosensing have been explored. Based on the above facts, this review also provides insights into the prevailing challenges and future prospects of the stimuli-responsive DNA hydrogels with an aim to promote the further development of the DNA hydrogels, especially in the field of biosensing.

## Strategies to fabricate DNA hydrogels

### Pure DNA hydrogels

**Fig. 1:**
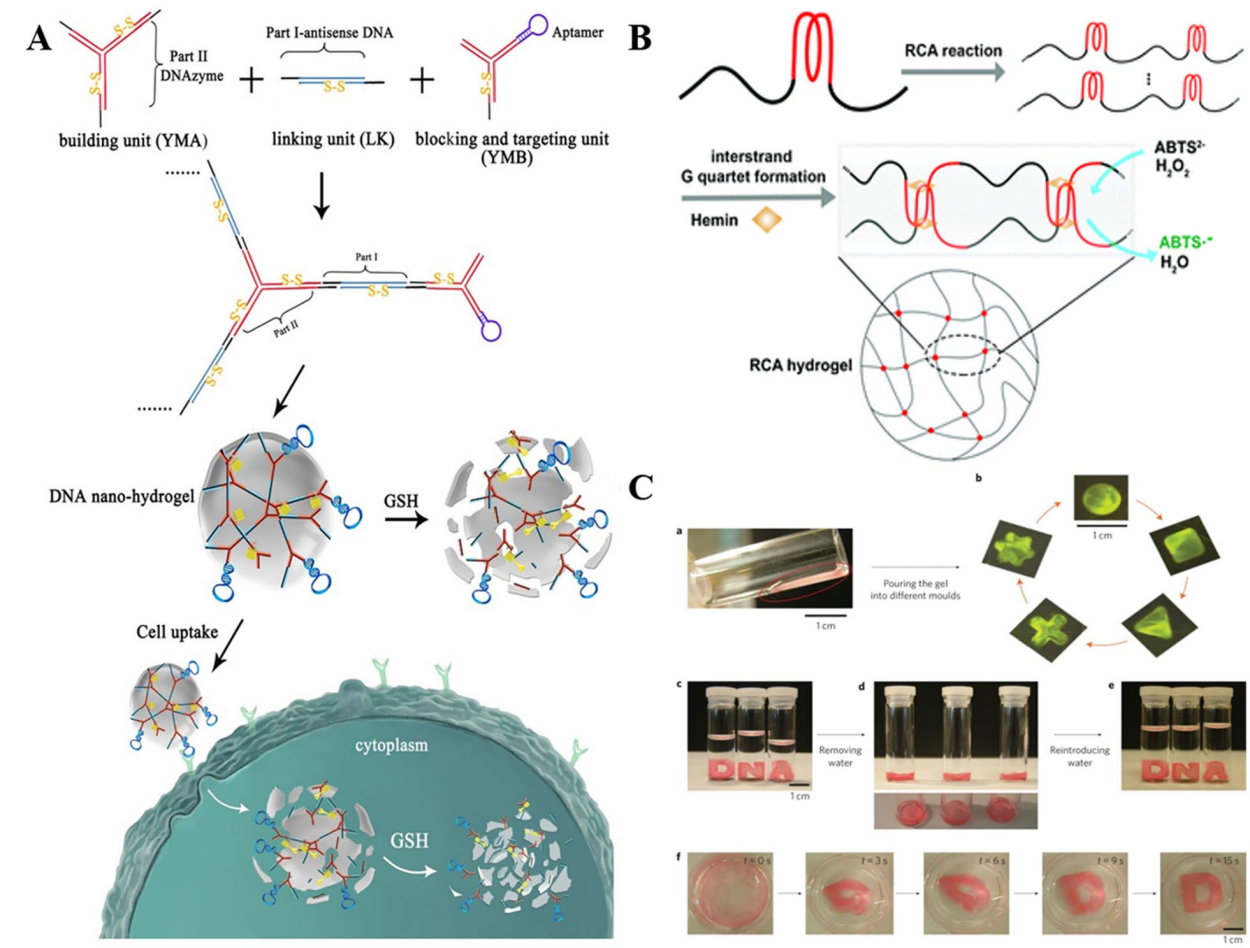
Examples of pure DNA hydrogels. **A** Schematic diagram of branched Y-shaped monomers (YMA and YMB) and linker that hybridize with their “sticky end” segments (black lines) to form nanohydrogel. Reproduced with permission from Ref. [61]. **B** Schematic diagram of the operation of the catalytic RCA hydrogel. Reproduced with permission from Ref. [65]. **C** When removing water, the hydrogels act as liquid and adapt to different containers. When reintroducing water, solid-like metaproperties of hydrogels occur. Reproduced with permission from Ref. [8]

The pure DNA hydrogels were first reported in 2006 [35]. Luo and colleagues designed three types of branched DNA monomers, namely X-DNA, Y-DNA, and T-DNA, with each sticky end of the monomers designed as palindromic sequences for hybridization and ligation by T4 ligase, in which the DNA monomers act as both cross-linking agents and cross-linking substrates. The properties of the hydrogel can be tuned by changing the concentration and type of initial DNA monomer to meet the needs of different applications. In addition to branching motifs alone, each type of branching motif can be hybridized with DNA linker double-stranded units. Liu et al. used double-stranded DNA (dsDNA) as a linker strand to ligate Y-monomers A and B to form a DNA hydrogel [61]. As shown in Fig. 1A, Y-monomer A (YMA) has three sticky ends for incorporation into deoxyribonucleases, Y-monomer B (YMB) has one sticky end for incorporation into the aptamer structure, and the linker chain has two sticky ends for incorporation into antisense oligonucleotides. The introduction of disulfide bonds allows the monomer and linker chains to remain stable in the blood circulation and to be cleaved by the reducing agent glutathione (GSH) upon reaching the targeted region, thus carrying out the release of the therapeutic gene or the loaded drug. Alternatively, without using branching motifs, separate linear dsDNA can be self-assembled as building blocks to form DNA hydrogels by rational design of the sticky ends [62]. Since DNA hydrogels are not covalently linked (by adding ligase to achieve this), they have temperature-responsive properties.

Owing to the inevitable accumulated errors associated with DNA self-assembly at high concentrations and cost constraints, the preparation of the pure DNA hydrogels by means of nucleic acid amplification has gained significant attention [8], mainly including hybridization chain reaction (HCR) [63], rolling circle amplification (RCA) [64] and multi-primed chain amplification (MCA). RCA represents a thermostatic amplification method. Using circular DNA as a template, dNTPs are ligated into long ssDNA by the DNA primers complementary to a portion of the circular template in the presence of polymerase, with the product containing the hundreds or thousands of repeats of the DNA fragment complementary to the template. Tian et al. developed an RCA method for an automated production of the DNA hydrogels and successfully used it to detect glucose [65]. As shown in Fig. 1B, long ssDNA with multiple repeats of the G-rich sequences is amplified by using a cyclic cytosine-rich DNA template. By the rational design of the template sequence, the resulting RCA product possesses an intermolecular four-stranded conformation, which provides a sufficient crosslinking force for hydrogel formation. The hydrogels exhibit a highly stable horseradish peroxidase-like (HRP) catalytic activity and can be maintained under harsh storage conditions for long term. This makes it promising for various applications in colorimetric analysis. Subsequently, the authors further designed three RCA templates to periodically align the I-motif-sequence forming sequences (IFSs) on the polymeric DNA strands by using the RCA method [66]. The generated RCA products are noted to readily form the intermolecular I-motif sequence structures between the DNA strands and are more likely to form the stable hydrogels in acidic environments, thus, opening the opportunities for the pH-stimulated drug release. Exceptionally, other studies have reported the combination of RCA and MCA amplification to prepare the DNA hydrogel metamaterials (termed as biased hydrogels) with unique mechanical properties [8]. As observed from Fig. 1C, the hydrogel possesses the liquid-like properties after removal from water and can adapt to the containers of different shapes. On the other hand, it exhibits the solid-like properties in water. Moreover, irrespective of the shape change, the hydrogel is noted to rapidly return to its original shape on adding water. The developed hydrogel with meta-properties exhibits high potential of use for drug release, cell therapy, electronic switches and flexible circuits.

### Hybrid DNA hydrogels

Although pure DNA hydrogels show some unique properties, there are still limitations. Intrinsic properties of DNA dictate high synthesis costs and a highly negative net charge, in addition to the few functionalities available in the DNA molecule, limiting further modifications and reducing versatility [67]. As a result, more and more researchers are turning to hybridized DNA hydrogels. Specifically, this class of DNA hydrogel is a gel material not composed entirely of DNA, it can refer to the binding of the DNA strands to different polymers and/or nanoparticles (plasmonic nanoparticles, magnetic nanoparticles and carbonaceous nanoparticles, etc.). The scope of the bio-responsive hybridized DNA hydrogels has been significantly expanded due to the introduction of the diverse functional groups, thus, providing additional molecular recognition capabilities and versatility. And the results of the comparison of the two different types of DNA hydrogels are summarized in Table 1.

**Table 1:** Comparison of the performance of typical DNA hydrogels

Category	Compound	Versatility	Biocompatibility	Adaptability	Biodegradability
Pure hydrogel	DNA	Low	High	Weak	Easy
Hybrid hydrogel	DNA + polymer/hybrids	High	Low	Strong	Hard

#### Polymer-grafted DNA hydrogels

The hybridized DNA hydrogels were first reported in 1996, and the DNA-polyacrylamide chain copolymer employed in the study has become one of the most established building blocks since then [60, 68–70]. Chiefly, two representative DNA hydrogels are prepared, one of them is formed by grafting two complementary DNA strands onto polyacrylamide strands separately and cross-linking them by base pairing. The other is the introduction of a linker chain, which is complementary to the modified DNA on each of the two linear strands, thereby crosslinking them to form DNA hydrogels.

In addition to the initially used double-stranded nucleic acids as crosslinking units, a rich “tool-box” of the structural motifs (I-motifs, G-tetramers, T-A·T/C-G·C triplets, C-Ag^+^-C, trans-azobenzene/β-cyclodextrin supramolecular complexes, glucosamine-boronic acid esters, etc.) has been proposed for crosslinking [71–76]. These motifs can not only crosslink individually to form the DNA hydrogels, but can also be synergistically cross-linked in different combinations, thereby imparting the dual/multi-trigger functionality and controlled rigidity to the hydrogels, resulting in a broad versatility [77].

**Fig. 2:**
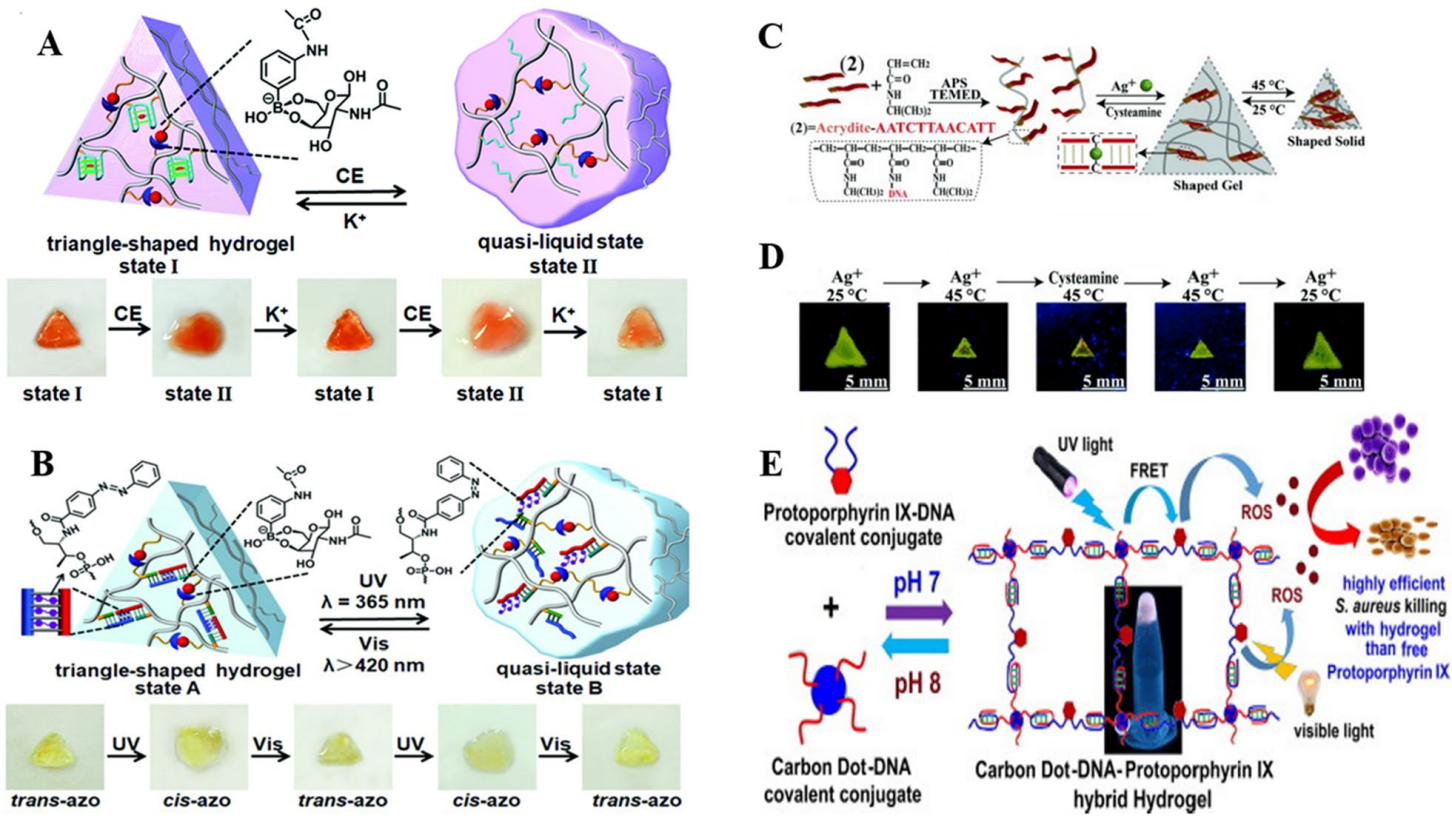
Schematic representation of the synthesis of hybrid DNA hydrogel. **A** Polyacrylamide DNA hydrogels of glucosamine-boronate eaters and G-quadruplexes synergistically cross-linked in the presence of K^+^/crown ethers undergoing cyclic hydrogel-solution transition. Reproduced with permission from Ref. [78]. **B** Shape-memory hydrogel crosslinked by glucosamine-boronate eaters and *trans*-azobenzene stabilized duplex. Reproduced with permission from Ref. [78]. **C** Reversible transformation of pNIPAM hydrogels with C-Ag^+^-C bridging and the corresponding images (**D**). Reproduced with permission from Ref. [79]. **E** The fabrication of DNA hydrogel cross-linked by covalent conjugation of DNA with fluorescent CD to accommodate PpIX as a photosensitizer for A-PDT applications. Reproduced with permission from Ref. [97]

Typically, in 2019, a novel hybrid DNA hydrogel was prepared by synergistically cross-linking an aminoglucose-borate (I) with a stimulus-responsive nucleic acid unit (II) [78]. As shown in Fig. 2A, based on the low-rigidity hydrogel formed by the cross-linked I chains, K^+^/crown ether allows the formation/dissociation of II so that the hydrogel cycles between high/low rigidity. Wherein, the I-strand provides an internal permanent memory and the external stimuli represented by K^+^/crown ether form a DNA gear that enables shape memory and self-healing functions. Similarly, the G-quadruplex can be replaced with other cross-linked units in the tool-box. As shown in Fig. 2B, the synergistic crosslinking unit is a double-stranded nucleic acid bridge stabilized by trans-azobenzene. It can be isomerized to the cis under 365 nm excitation light, while returning to the trans state under > 420 nm excitation, realizing the photoisomerization switch. Compared to the traditional absolute 0-1 switching of hydrogels under a single trigger, the presence of a quasi-liquid state (0.5) provides a temporary shape (storage), and the orthogonal trigger function gives it more possibilities in developing stimulus-responsive “actuators”.

In addition to polyacrylamide, different polymer backbones have been continuously explored, bringing different stimulus-responsive properties. A relatively typical one is the temperature-responsive DNA poly-N-isopropylacrylamide (pNIPAM) based hydrogel [79]. It exhibits a reversible gel-to-solid transition occurs at approximately 32 °C [80]. While the incorporation of metal ions (e.g., Cu^2+^, Hg^2+^, Ag^+^, etc.) or photoisomerization groups tethered to the polymer can alter the gel-to-solid transition temperature of the cross-linked polymer [81, 82]. In 2014, Guo et al. developed a dual-trigger thermally responsive DNA hydrogel using pH or metal ions/ligands as co-triggers for the polymer phase transition [79]. This is exemplified in Fig. 2C. Under the stabilization of C-Ag^+^-C complexes, dsDNA bridges NIPAM to form DNA hydrogels (G′ = 85 Pa, G′′ = 4 Pa). Whereas, after the presence of cysteamine eliminates the effect of Ag^+^, the hydrogel reverses to dissolve into a liquid. Heating the triangular DNA hydrogel formed in the Teflon mold to 45 °C yields a compressed triangular structure that expands back to the hydrogel state upon cooling. Notably, the solid structures heated to 45 °C do not dissociate into solution under the influence of cysteamine (Fig. 2D). Likewise, DNA hydrogels formed by cross-linking DNA using C-rich sequence oligonucleotide tether strands under acidification conditions (pH = 5.2) degrade to liquid after neutralization (pH = 7.2) and undergo a reversible solid-hydrogel transition when treated at 45 °C and 25 °C.

#### DNA hydrogels based on other hybrids

In addition to the polymeric backbones, a few research studies have also introduced components such as nanoparticles [83–86], proteins [87], quantum dots (QDs) [88], carbon nanotubes (CNTs) [89, 90], metal organic frameworks (MOFs) [91, 92], silver nanoclusters [93], graphene [94, 95] and magnetic materials [96] in the pure DNA hydrogels in order to confer the hybrid hydrogels additional functionality and versatility. In 2019, Sonam Kumari and coworkers reported a novel smart DNA hydrogel [97]. As shown in Fig. 2E, carbon dots (CDs) and protoporphyrin IX (PpIX) are attached to the ends of ssDNA strands through covalent bonds, respectively, and the coupled DNA forms DNA hydrogels by forming I-motif structure. I-motif structure can respond to pH for gel-sol transition. And the unique photophysical properties of CD and PpIX allow tracking of the release of loaded PpIX and degradation of the hydrogel. Recently, Yang’s group used DNA hydrogels formed by physical entanglement after RCA reaction as a binding scaffold for silver nanoclusters to obtain multifunctional hybrid hydrogels [93]. The unique mechanical properties of DNA hydrogels formed by this polymerization method (relayed RCA) make it injectable. And the presence of silver nanoclusters gives DNA hydrogels excellent fluorescence properties and strong antibacterial activity, facilitating their application in biomedical fields such as tissue engineering, wound dressing, biosensing and bioimaging. Single-walled carbon nanotubes (SWCNTs) exhibit strong near-infrared light absorption, ultra-high electrical and thermal conductivity. Based on this, Nikhita D. Mansukhani and colleagues combined them with DNA to form hybrid DNA hydrogels [89]. The modulation of the DNA hydrogels mechanical properties was achieved by varying the ratio of SWCNTs to DNA, and the formed DNA hydrogels are also photo-reversible and thermally reversible, which can be triggered by temperature changes or near-infrared radiation. The hydrogels have a wide range of promising applications including substrate corresponding materials, sensing and 3D printing. In particular, Li and his colleagues reported the preparation of double-network hybrid hydrogels by a simple “one-pot” mixing method [98]. The hydrogels consist of networks formed by two hydrogel systems interpenetrating each other, one using DNA hybridization and the other using host-guest interactions of cucurbit [8] uril (CB [8]). The interpenetration of the well homogeneous network leads to a nonlinear increase in mechanical strength and crack resistance. In addition, the hydrogels are fully biodegradable, consisting of nuclease degradation of the DNA network and cellulase cleavage of the CB [8] network. Based on these excellent properties, this supramolecular hydrogel network can be used as a novel soft material scaffold for dynamic surface coating, controlled release and tissue engineering applications.

## DNA hydrogels for biosensing applications

The response of the stimuli-responsive DNA hydrogels to external stimuli is widely used in many fields, especially in biosensing [95]. The biosensors are the unique sensing devices that convert the biological substances into acoustic, optical and electrical signals for detection. The incorporation of DNA in the hydrogel biosensors introduces additional interfaces, thus, DNA acts both as a cross-linking unit and as a recognition element. The specific recognition of the analyte triggers a change in the mechanical properties of the hydrogel matrix, which can be further converted into a signal suitable for detection. In the following section, the specific applications of the DNA hydrogels in biosensing have been discussed with respect to the different output signals, such as optical, electrochemical, point-of-care testing (POCT) etc. These applications are summarized in Table 2.

**Table 2:** Stimuli-based DNA hydrogels based biosensing strategies

Output signal	Type of DNA hydrogel	Target	Sensitivity (LOD)	Detection range	Detection time	RSD (%)	Ref
Fluorescence	Hybrid	miR-141	10^–4^ fM	10^−4^fM-10 pM	N/A	4.3	[99]
Fluorescence	Hybrid	miR-141	1.1 × 10^–4^ fM	10^–3^ fM-10 pM	N/A	5.2	[99]
Fluorescence	Hybrid	OTA	0.01 ng mL^−1^	0.05–100 ng mL^−1^	2 h	4.26	[101]
Fluorescence	Hybrid	AIV H5N1	0.4 HAU	2^–1.2^–2^6^ HAU 20 μL^−1^	30 min	N/A	[103]
Fluorescence	Hybrid	DNA	6 fM	6 × 10^–15^-6 × 10^–9^ M	1 h	N/A	[104]
Colorimetric	Hybrid	Glucose	0.44 mM	0–15 mM	1.5 h	N/A	[108]
Colorimetric	Hybrid	Pb^2+^	13.9 nM	50–700 nM	1 h	N/A	[110]
Colorimetric	Hybrid	T-2 toxin	0.87 pg mL^−1^	0.01–10,000 ng mL^−1^	1.5 h	3.87/7.07	[112]
Colorimetric	Hybrid	MC-LR	3 ng L^−1^	4.0–10,000 ng L^−1^	N/A	≤ 5.52	[116]
Colorimetric	Hybrid	H_2_O_2_	1 μM	N/A	1 h	N/A	[117]
Colorimetric	Pure	Ct DNA	0.32 pM	1 pM-10 nM	N/A	3.63/6.15	[118]
SERS	Hybrid	AFP	50 pg mL^−1^	50 pg mL^−1^–0.5 μg mL^−1^	2 h	0.9/1.7	[56]
SERS	Hybrid	UO_2_^2+^	0.838 pM	1 pM-0.1 μM	2 h	N/A	[128]
SERS	Hybrid	miR-155	0.083 fM	0.1 fM-100 pM	40 min	0.8/2.6	[129]
SERS	Hybrid	miR-122	7.75 aM	10 aM-100 pM	N/A	0.59	[130]
SPR	Hybrid	PML/RARα	45.22 fM	100 fM-10 nM	N/A	5.29/7.71	[134]
CL	Hybrid	D-Dimer	53.7 fg mL^−1^	100 fg mL^−1^–100 ng mL^−1^	3 h	2.96/4.51	[55]
CL	Hybrid	FDP	31.6 fg mL^−1^	100 fg mL^−1^–100 ng mL^−1^	3 h	3.73/4.76	[55]
CL	Hybrid	Adenosine	1.04 × 10^–13^ M	4 × 10^–13^-1.5 × 10^–10^ M	N/A	2.8	[135]
ECL	Hybrid	Let-7a	1.49 fM	10 fM-10 nM	N/A	0.59/1.13	[54]
Electrochemical	Hybrid	miR-21	5 nM	10 nM-50 μM	N/A	N/A	[139]
Electrochemical	Pure	H_2_O_2_	13 nM	30 nM-100 μM	N/A	2.4	[140]
Electrochemical	Hybrid	HPA	0.003 pg mL^−1^	0.01 pg mL^−1^–20 ng mL^−1^	2 h	7.36/9.35	[142]
Electrochemical	Hybrid	Hg^2+^	0.042 pM	0.1 pM-10 nM	N/A	3.2/6.8	[143]
Glucometer	Hybrid	miRNAs	0.325 fM	0.5–250 fM	N/A	N/A	[50]
Glucometer	Hybrid	Adenosine	1.6 μM	0–750 μM	2 h	N/A	[70]
Glucometer	Hybrid	Dam	0.001 U mL^−1^	0.001–5.0 U mL^−1^	1 h	N/A	[149]
V-chip	Hybrid	Adenosine	0.06 μM	0–1 μM	30 min	N/A	[150]
V-chip	Hybrid	OTA	1.27 nM	0–1 μM	1.5 h	N/A	[151]
V-chip	Hybrid	Uranium	37 nM	0–600 nM	N/A	N/A	[164]
Pressuremeter	Hybrid	Cocaine	0.12 μM	0–400 μM	15 min	N/A	[153]
Electronic balances	Hybrid	AFB1	9.4 μg kg^−1^	31.2 μg kg^−1^–6.2 mg kg^−1^	30 min	1.3/4.9	[69]
pH meter	Hybrid	AFB1	0.1 µM	0.2–20 µM	60 min	N/A	[165]
Microfluidic	Pure	MERS	0.1 × 10^–12^ M	N/A	30 min	N/A	[157]
Microfluidic	Pure	Pathogen	0.019 pM	N/A	15 min	N/A	[158]
Microfluidic	Pure	SARS-CoV-2	0.7 aM	N/A	15 min	N/A	[166]
µPADs	Hybrid	Cocaine	3.8 μM	10–400 μM	1.5 h	N/A	[162]
µPADs	Hybrid	Cocaine	4.5 μM	0–500 μM	N/A	N/A	[163]
LF-NMR	Hybrid	BPA	0.07 ng mL^−1^	10^–2^-10^2^ ng mL^−1^	N/A	N/A	[86]
PEC	Hybrid	p53	20 fM	100 fM-10 nM	60 min	N/A	[51]
Capillary	Hybrid	Pb^2+^	10 nM	0.01–50 μM	1 h	4.6	[167]
Ellipsometry	Pure	Let-7a	0.2 fM	0.2–500 fM	4 h–6 h	N/A	[53]
Ellipsometry	Pure	miR-375	10 fM	0.01–700 pM	4 h–6 h	N/A	[53]
Ellipsometry	Pure	miR-21	40 pM	0.04–100 nM	4 h–6 h	N/A	[53]

### DNA hydrogels in optical biosensing

#### DNA hydrogels in photoluminescent biosensing

Generally, there are two mechanisms for constructing DNA hydrogel-based fluorescent sensors. The first type is the modification or encapsulation of fluorophores (SYBR Green or other fluorescent indicators) in DNA hydrogels, and the second type is based on the “ON/OFF” mechanism of fluorescence resonance energy transfer (FRET), where bursts occur during assembly due to proximity and alteration upon recognition of a specific event.

**Fig. 3:**
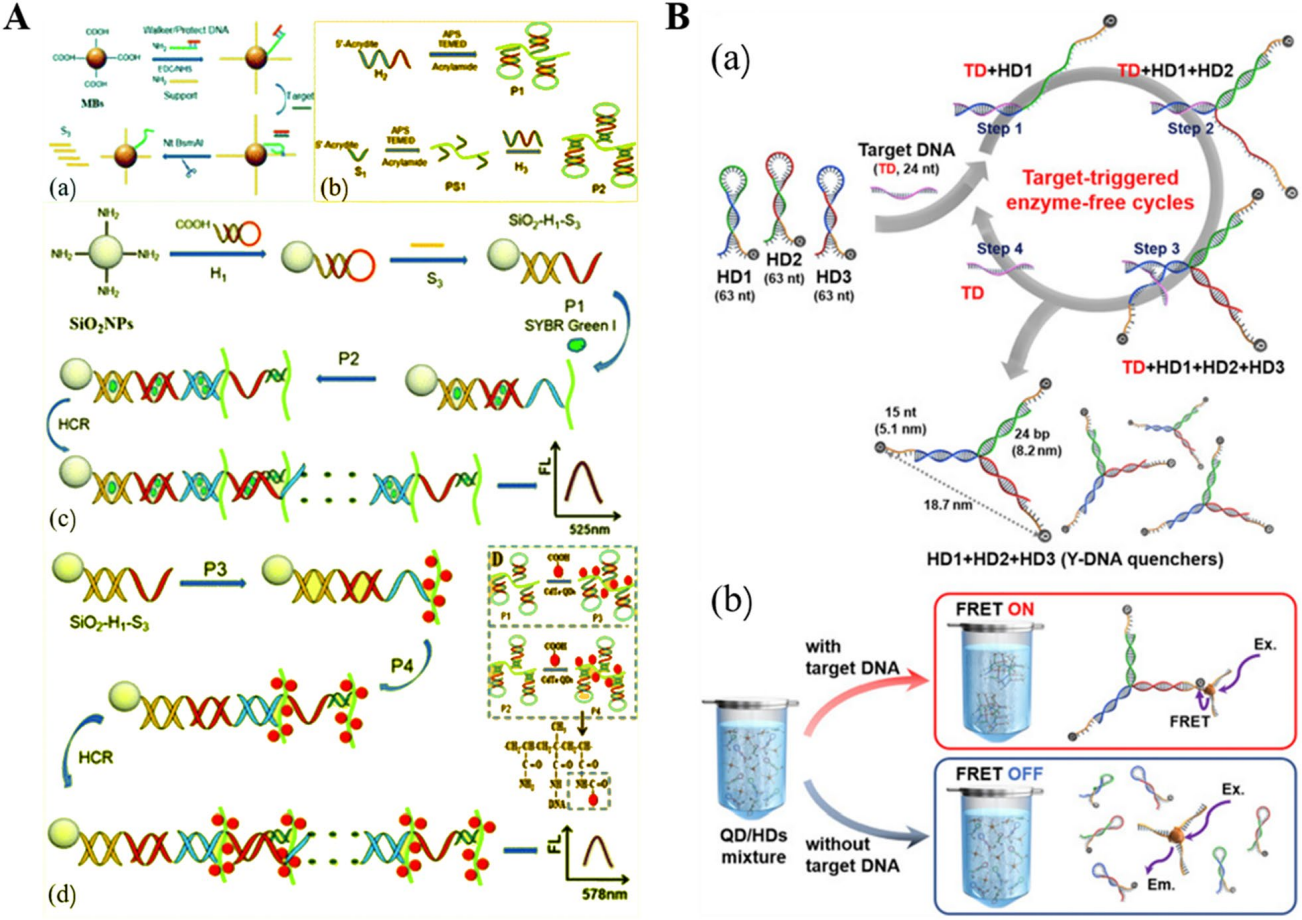
Schematic illustration of stimuli-responsive hydrogel-based photoluminescent biosensing strategies. **A** Fabrication of the DNA hydrogel fluorescence platform for detection of target miRNA. Reproduced with permission from Ref. [99]. **B** Schematic diagram of the QD-DNA hydrogel based on FRET detection of DNA. Reproduced with permission from Ref. [104]

In 2019, Li et al. reported a DNA hydrogel fluorescence detection platform constructed by using DNA Walker and HCR amplification [99]. As shown in Fig. 3A, DNA Walker amplifies a large number of single-stranded S3 in the presence of target miRNAs, and after base sequence design, S3 can bind to hairpin DNA (H2 and H3) sequences to trigger HCR amplification and form DNA cross-linked hydrogels. Extensive multi-branched dsDNA generated during amplification offers the opportunity to load SYBR Green I dye and CdTe QDs, resulting in a markedly amplified fluorescent signal and obtaining a 10^–4^ fM level of detection limit in the detection of human prostate cancer miR-141. After that, Kunfu Pi et al. constructed a green fluorescent hydrogel sensor and applied it in practice [100]. The authors take advantage of the selective binding of Hg^2+^ to thymine, the formed T-Hg^2+^-T crosslinker can bind SYBR Green I dye. After performance optimization the monitoring of Hg^2+^ in water (even in complex freshwater environments such as the Lawrence Great Lakes) was achieved. In 2020, Wu and coworkers were the first to integrate competitive binding patterns of aptamers, complementary sequences and targets into RCA self-assembled DNA hydrogels to construct DNA hydrogel-based fluorescent sensors that can be used for food safety monitoring sensitively [101]. The presence of the target dissociates the primers for the RCA reaction, and the released primers hybridizes with padlock probes to form a circular template, which is amplified with the assistance of polymerase, ligase, and Cy3-dNTPs to form a fluorescent DNA hydrogel. The fluorescence intensity is positively correlated with the concentration of the target and has excellent specificity and stability.

In addition to modifying the fluorophores on the cross-linked strands, the three-dimensional porous structure of the DNA hydrogel allows for the encapsulation of the fluorophore with no additional modification steps. Xiang et al. used X-DNA as the building block, terminal deoxynucleotidyl transferase (TdT) as the “glue” and dATPs/dTTPs as the ingredients to extend and hybridize to form DNA hydrogels [102]. Prior incorporation of green fluorescent protein allows it to be encapsulated into the matrix and its release kinetics are monitored within 10 h after the addition of DNA endonuclease I (DNase I) for cleavage of DNA hydrogels.

Besides fluorescent indicators, Xu et al. reported a label-free DNA hydrogel fluorescent sensor with FRET signal output, enabling the detection of avian influenza virus H5N1 [103]. The authors label the QDs and quenchers on the complementary aptamer and single chain, respectively, and the fluorescence of the QDs is burst due to the proximity during cross-linking. In the presence of the target, the aptamer dissociates, the fluorescence donor-acceptor distance increases, and fluorescence is restored. The constructed method can achieve the detection of the target within 30 min and has potential for application in on-site detection. In 2021, Cheol Am Hong et al. constructed an enzyme-free DNA hydrogel based on the FRET mechanism to achieve rapid (1 h) and highly sensitive (fM level) detection of target DNA [104]. As shown in Fig. 3B, three individual hairpin DNAs (HD1, HD2, HD3) are designed with the 3′ end of each hairpin labeled with the quenchers BHQ-2. In the presence of the target DNA, the hairpin successively opened and eventually a Y-nanostructure (fluorescent burst) is obtained. The released target DNA can repeatedly open another HD1 and catalyze the loop-opening cycle, producing a large amount of Y-DNA with no need for enzymatic catalysis. In this regard, Y-DNA can be assembled with QD functionalized with complementary ssDNA to form QD-DNA hydrogel, and FRET is in “ON” state, thus reducing the fluorescence intensity. In the absence of target DNA, there is no effect on FRET and it is in the “OFF” state. The constructed method can provide an alternative method for the detection of short DNA sequences with low abundance.

The DNA hydrogel-based fluorescence detection method is easy to operate, the detection limit is satisfactory, and the reliability of the assay will be greatly improved if the background noise can be effectively reduced.

#### Application of DNA hydrogels in colorimetric biosensing

The colorimetric assay has many strengths such as simplicity of operation, cost-effectiveness, fast response time, and the fact that the signal can be output by a simple portable spectrometer or image processing software, even with the naked eye [105]. DNA hydrogels, due to its optical transparency, allow for the standard staining and labeling to perform colorimetric analytical assays, therefore, many efforts have been made in the development of colorimetric sensors.

**Fig. 4:**
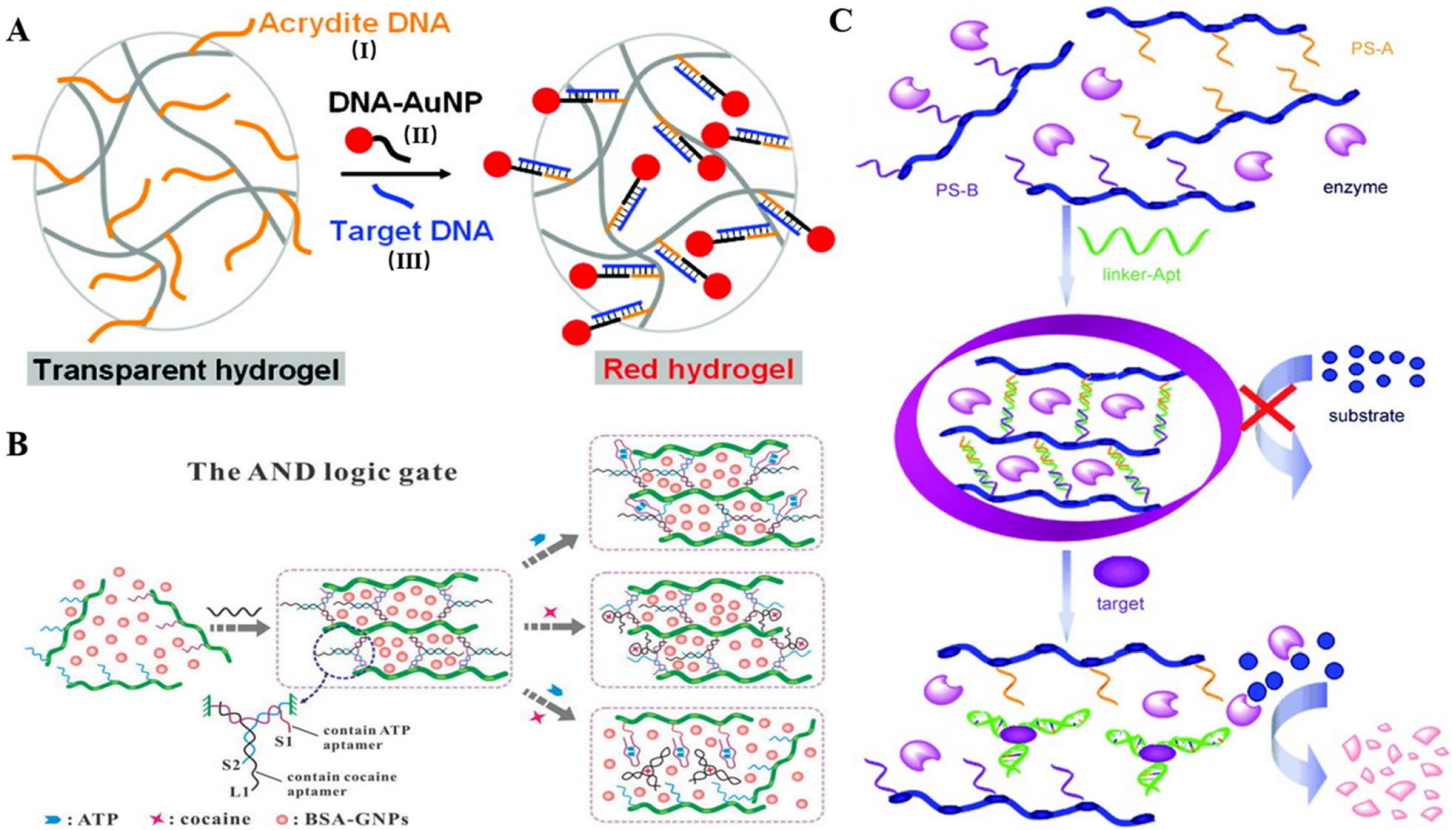
Examples of stimuli-responsive DNA hydrogel-based colorimetric detection. **A** Preparation of DNA-functionalized monolithic hydrogels and AuNPs as color indicator. Reproduced with permission from Ref. [107]. **B** Fabrication of AND logic gate for cocaine and ATP detection. Reproduced with permission from Ref. [109]. **C** Enzyme trapped hydrogel for signal amplification and visual detection. Reproduced with permission from Ref. [46]

The extinction coefficient of gold nanoparticles (AuNPs) at visible wavelengths (~ 520 nm) is very large, which makes them a sensitive indicator for visual detection [106]. The DNA hydrogels allow modification of DNA and thiol-modified DNA can bind to AuNPs by thiol-gold interaction, based on which Ajfan Baeissa prepared DNA functionalized monolithic hydrogels [107]. As shown in Fig. 4A, DNA (I) is modified on an acrylamide strand and another DNA (II) containing a 3′-thiol modification that can be ligated to AuNPs. In the presence of the target DNA (III), I and II can be linked, thus attaching AuNPs to the surface of the gel and thus allowing visual detection without any instrumentation. Additionally, the high loading of DNA hydrogels encapsulating AuNPs can be used for detection. DNA hydrogels can physically isolate the AuNPs from the solution and un-isolate it after the stimulus response. Undoubtedly, the high loading and stimulus response characteristics bring about an increase in sensitivity and specificity. In 2018, Ma and colleagues encapsulated AuNPs in aptamer cross-linked hydrogels for colorimetric detection of glucose [108]. The aptamer as a cross-linked chain recognizes the glucose complex and dissociates from the complementary single chain, causing the hydrogel to disintegrate and releasing the encapsulant for visual colorimetric detection. Combining aptamer cross-linked hydrogels and smart materials, Yin et al. developed a chemical stimulus-controlled colorimetric logic gate system (“AND” and “OR”) [109]. Notably, in the “AND” logic gate (Fig. 4B), the DNA hydrogel is cross-linked in the form of a “Y” structure with two aptamers cross-linked to the linker chain, with each strand containing two semi-complementary structural domains of the other two strands. The addition of less than or equal to one target don’t disable the linkage structure, and only when two targets are present at the same time, the Y-type structure dissociates and cannot maintain the hydrogel stability and releases AuNPs for colorimetric detection. This method is expected to provide a universal detection platform for polymeric logic gates by simply designing functional nucleic acids for programming. In addition to the difference in color intensity produced by the change in AuNPs concentration, Mao et al. used DNA hydrogel-coated gold nanorods to achieve color chromaticity change as the output signal for the detection [110]. Since gold nanorods have different colors at different aspect ratios, reactive radicals generated by different concentrations of the target can etch them, thus changing the aspect ratio and enabling visual detection.

Encapsulating colored substances in DNA hydrogel for direct detection based on stimulus-responsiveness, which is simple and fast. However, the sensitivity and quantification issues still limit its application. To address this issue, enzymes and other bioactive materials are usually introduced [111]. Due to the inherent biocompatibility, programmability, and high loading capacity of DNA hydrogels, they are ideal scaffolding materials for a variety of chromogenic reaction substrates.

In 2009, Zhu et al. first combined a DNA hydrogel with an amylose-I_2_-amylase system for the visual detection of cocaine [46]. As Fig. 4C, the DNA hydrogel is coated with amylase, and then the amylose is mixed with a small amount of iodine solution, at which time the color is dark blue. After adding the target cocaine to the reaction system, the aptamer as a cross-linker can combine with cocaine and release amylase, which can catalyze the decomposition of amylose into sugar, at which time the color changes to colorless, thus realizing naked eye detection. This strategy allows the detection of < 20 mg of cocaine in 10 min, and the detection of other targets can be achieved by replacing the aptamer crosslinking chain with simplicity and versatility. Recently, Sun et al. achieved the detection of T-2 toxin using DNA hydrogels coated with HRP [112]. HRP can catalyze the reaction of H_2_O_2_ with KI to form I_2_, resulting in a color change by longitudinally etching gold nanorods in the presence of high concentrations of cetyltrimethylammonium bromide (CTAB). The LOD of this aptamer hydrogel sensor is 0.87 pg mL^−1^, which is lower than that of other conventional methods. In fact, natural enzymes are susceptible to environmental factors in use, so nanocomposites that mimic natural enzymes have been developed rapidly [113–115]. Recently, Wu et al. combined DNA hydrogels with the peroxidase activity of Cu/Au/Pt trimetallic nanoparticles to achieve the quantitative detection of microcystin-LR (MC-LR) [116]. Solubilization of DNA hydrogels by competitive binding of MC-LR to aptamers releases preloaded nanoparticles. The nanoparticles show greater catalytic activity than mono- or bimetals and catalyzes the production of oxTMB products visible to the naked eye from the substrate 3,3,5,5-tetramethylbenzidine (TMB) in the presence of H_2_O_2_. Based on this sensitive strategy, a detection limit of 3 ng L^−1^ is achieved, offering the possibility to analyze actual samples.

Functional nucleic acid DNAzyme, which can be assembled in the hydrogel network as a cross-linker, represents a class of catalytic DNA structures that can mimic the function of natural enzymes, has the advantages of low preparation cost and good thermal stability [117]. For example, Mao et al. prepared a DNA hydrogel with target DNA triggering and DNAzyme functionalization [118]. By sequence design of the padlock template of the target DNA, the macroscopic hydrogel formed by the triggered RCA amplification can be made to have a degree of G-quadruplex structure. Upon addition of hemin, the DNAzyme activity of G-tetramer/hemin can catalyze the oxidation of the colorless substrate 2,2′-azino-bis (3-ethyl-benzothiozoline-6-sulfonic acid) (ABTS) to green ABTS*, achieving a detection limit of 0.32 pM with no complex equipment. The constructed strategy has excellent accuracy and reliability, which is expected to realize the application in remote areas. DNAzyme also allows DNA self-cleavage reactions assisted by the metal ions, of which metal ions are indispensable cofactors, which provides the possibility of their detection. Lin et al. used Cu^2+^-dependent DNAzyme/substrate complexes to cross-link linear polymers into hydrogels for the detection of Cu^2+^ [119]. AuNPs are preloaded in hydrogels as colorimetric indicators, and the addition of metal ions activates the DNAzyme, which then undergo irreversible cleavage reactions at the cleavage sites, thus bypassing the competitive mechanism of stoichiometric ratios, improving sensitivity, and with excellent specificity. Similar methods can also be used for the detection of Ln^3+^ [120].

Clustered regularly interspaced short palindromic repeats (CRISPR) and CRISPR-associated (Cas) nucleases, the adaptive immune system of microorganisms, have revolutionized the field of biomedicine since their discovery [121]. Besides being widely used as a gene editing tool, it also has a promising development in the field of nucleic acid detection [122–125]. Cas12a displays specific cleavage activity for RNA-directed dsDNA (targeted cleavage) and efficient cleavage of nearby ssDNA with non-specific multi-turnover upon activation (lateral cleavage) [126]. DNA-responsive hydrogels use ssDNA as a crosslinker and are ideally suited to dock with the non-specific cleavage activity of Cas12a. The responsive properties of conventional DNA hydrogels rely on strand displacement or structural changes of DNA cross-linkers, which may require higher target concentrations. In contrast, side-branch cleavage by Cas12a enzymes can control the properties of DNA hydrogels in a modular fashion at multiple scales, thereby effectively translating external stimuli into changes in material properties upon signal amplification. In 2020, Max A. English and colleagues combined the DNA hydrogels with Cas12a nuclease to enable colorimetric detection [126]. DNA-polyacrylamide hydrogels pre-loaded with AuNPs are synthesized, in which the cross-linked strands contain AT-rich Cas12a lateral branch cleavage sites. When the hydrogel is exposed to Cas12a and triggers dsDNA, the bridging strand is cleaved and the gel degrades thereby releasing AuNPs to achieve colorimetric detection of dsDNA. This platform offers improved programmability and increased sensitivity of hydrogels, showing promise for a wide range of applications.

#### DNA hydrogels in surface-enhanced Raman spectroscopy (SERS) biosensing

Due to the advantages of non-destructive detection, high sensitivity, biocompatibility and specificity, SERS has been widely investigated in the field of biosensing [127]. The switchable DNA hydrogel allows for SERS signal flexibility, and the incorporation of DNA not only delivers stimulus response characteristics, but also allows for signal amplification in combination with amplification means.

Recently, Wang et al. combined the advantages of SERS with stimulus-responsive DNA hydrogels for the detection of α-fetoprotein (AFP) [56]. Immunoglobulin IgG is encapsulated in an AFP cross-linked DNA hydrogel, and upon introduction of AFP, the aptamer binds to it causing the hydrogel to disintegrate and IgG to be released. Subsequently, in the solution, the SERS probe, biofunctional magnetic beads can form a sandwich structure with IgG, and the Raman signal of the supernatant is reduced after magnetic separation. A higher concentration of AFP releases more IgG, more SERS probes bound, and weaker Raman signal obtained for quantitative detection of AFP. In addition, He and coworkers constructed a flexible SERS sensor based on DNAzyme hydrogels for UO_2_^2+^ detection [128]. The UO_2_^2+^-dependent DNAzyme is used as a cross-linker to form a hydrogel encapsulating the Raman probe RhB, at which point the Raman signal is negligible. Upon addition of the target, the DNAzyme complex responds by dissociating the hydrogel, and the released Raman probe can generate Raman signal, enabling the specific detection of UO_2_^2+^.

**Fig. 5:**
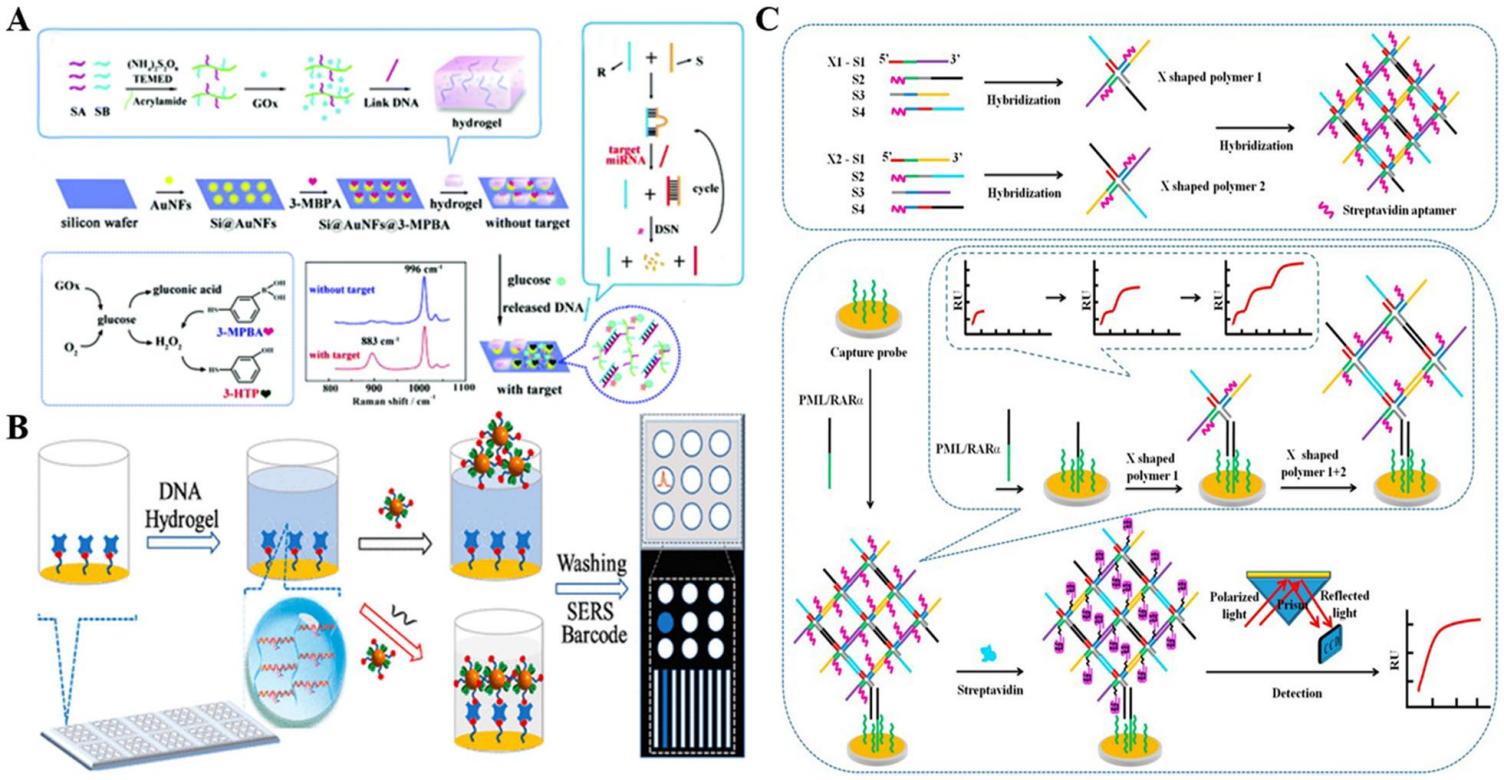
Examples of stimuli-responsive DNA hydrogel-based other optical detection. **A** Mechanism of the rationmatric SERS biosensing strategy via target-responsive DNA hydrogel. Reproduced with permission from Ref. [130]. **B** Schematic of multiple miRNAs detection in one sample using DNA hydrogel-based SERS biosensor array. Reproduced with permission from Ref. [131]. **C** Schematic diagram of the SA encapsulated DNA hydrogel-based SPR biosensing strategy for PML/RARα detection. Reproduced with permission from Ref. [134]

Combined with a signal amplification strategy, He et al. constructed a switchable target-responsive DNA hydrogel SERS sensing platform to achieve sensitive detection of miRNA-155 [129]. As shown in the Fig. 5A, the Raman reporter molecule toluidine blue (TB) is encapsulated in an L1 cross-linked DNA hydrogel, physically isolated from the substrate. The R1 sequence, which is complementary to L1, is designed to act as a “key” to turn on the switch for cutting the DNA hydrogel, and the S1 and R1 partly hybridizes to act as a “magic box” so that R1 could not function. In the absence of the target RNA, it shows a blind Raman signal in the off state. When the target miRNA-155 is present, the RNA dissociates from R1 by fully complementary hybridization with S1, after which the hybrid is cleaved by specific double-stranded nuclease (DSN) to regenerate the target RNA and enters the next cycle, thus releasing a large amount of R1. When the mixture containing multiplicative R1 is mixed with the gel, L1 can bind with R1 to open the three-dimensional structure and TB molecules are released, generating a strong Raman signal for quantitative detection. Based on this label-free, high-sensitivity detection principle, the platform achieves a detection limit of 0.083 fM and a linear range of 0.1 fM-100 pM, which is promising for clinical applications. Later, in 2019, He and colleagues constructed DNA hydrogel-based ratiometric SERS sensor that further improved the detection sensitivity [130]. In this paper, 3-mercaptophenylboronic acid (3-MPBA) is used as a SERS substrate, which can efficiently and specifically react with H_2_O_2_ to produce the corresponding phenol. The borate-to-phenol reaction produces a Raman peak of 883 cm^−1^, while the Raman peak of the substrate itself of 996 cm^−1^ remains essentially unchanged during this conversion, thus allowing for ratiometric measurements. Glucose oxidase (GOx) is encapsulated in the linker cross-linked DNA hydrogel, and when the target RNA appears, it will dissociate R in the same cyclic steps as above, and the dissociated R strand can disintegrate the hydrogel by hybridizing with the hydrogel linker, and Gox is released to catalyze the production of H_2_O_2_ from glucose, and the resulting H_2_O_2_ reacts with 3-MPBA to produce 3-hydroxyphenol (3-HTP). In this way, the peak intensity ratio of 883 cm^−1^ to 996 cm^−1^ is proportional to the amount of target RNA, achieving a detection limit of 7.75 aM and providing an alternative method for highly sensitive and accurate detection of trace amounts of the target. Recently, Si et al. achieved high-throughput target detection based on the SERS sensor array [131]. As shown in Fig. 5B, the array consists of nine sensing units with streptavidin (SA)-modified sensor units enclosed in switchable DNA hydrogels, at which point the SERS tags (biotin/4-mercaptobenzoni-trile-functionalized AuAg alloy nanoparticles (B/M-AuAgNPs)) cannot bind to them, showing a blank or weak Raman signal. After the introduction of the target RNA, the DNA hydrogel of the corresponding sensing unit is disintegrated and the SERS tag can bind to the SA-modified sensor surface through the dissolved liquid-phase hydrogel, generating a strong raman signal, thus enabling the detection of multiple targets in a sample.

#### DNA hydrogels in other optical analysis

Surface plasmon resonance (SPR) is a label-free, real-time analytical method that enables high sensitivity detection while being fast and efficient [132, 133]. DNA hydrogels can enhance the SPR signal significantly by recognizing and encapsulating large molecular weight substances to form supramolecular weight nanostructures. Recently, Guo et al. used a DNA self-assembling aptamer hydrogel-coated SA for signal amplification to achieve label-free, enzyme-free, ultrasensitive detection of the fusion gene PML/RARα with SPR as the output signal [134]. As shown in the Fig. 5C, two types of building blocks X1 and X2 are prepared by sequence design, both of which have SA aptamer sequences at the 5′ end for capturing SA and complementary sticky ends at the 3′ end for mutual binding. After adding the target, the capture probe immobilized on the chip surface hybridizes with it, and the exposed fragment of the target can hybridize with X1, which then hybridize with X2, and cross-linked sequentially to finally form an aptamer-based networked hydrogel nanostructure. The aptamer can bind a large amount of SA and further amplify the reaction signal. And in the absence of the target, the hydrogel structure cannot be formed and the background signal is negligible. The SPR sensor enables fM level detection of PML/RARα with a linear range of 100 fM-10 nM, and the excellent performance make it possible to provide a platform alternative for efficient detection of multiple fusion genes in clinical diagnosis.

Chemiluminescence (CL) method offers the advantages of no external light source, high sensitivity and simple operation. Recently, Lin et al. used target-responsive hydrogels coated with Au@HKUST-1 to form a CL biosensor for adenosine-specific detection [135]. The ssDNA, hemin aptamer, and adenosine aptamer are complementary to each other and cross-linked to form a DNA hydrogel. The hydrogel forms an "AND" logic gate and only when both adenosine and hemin are present, the hydrogel will dissociate to release the encapsulated Au@HKUST-1. In this case, the formed G-quadruplex/hemin and Au@HKUST-1, which enter the CL system, can achieve dual signal amplification of CL. This CL sensor can detect adenosine down to 1.04 × 10^–13^ mol L^−1^ and can provide an alternative method for adenosine detection. The CL system with long afterglow can maintain a stable signal intensity during analysis and is a hot research topic in this field [136, 137]. The dense structure and mechanical rigidity of DNA hydrogels cause a confinement effect that effectively retards the diffusion of reactants, resulting in long-lasting CL. For example, Wu and coworkers constructed a glow-type CL system based on DNA hydrogels and realized biosensing applications [55].

Recently, Zhu et al. reported a DNA hydrogel-based electrochemiluminescent (ECL) sensing strategy for miRNA detection [54]. This strategy utilizes the strong affinity and good ECL properties of amphiphilic pyrene derivative (PTC-DEDA) as DNA embedding agent and ECL luminescent. In the presence of the target miRNA, a nonlinear chain hybridization reaction is induced to generate DNA hydrogels in situ, which can be loaded with large amounts of PTC-DEDA to achieve a strong ECL signal. The constructed DNA hydrogel biosensing platform provides an ECL analysis method with an LOD of 1.49 fM.

### DNA hydrogels in electrochemical biosensing

**Fig. 6:**
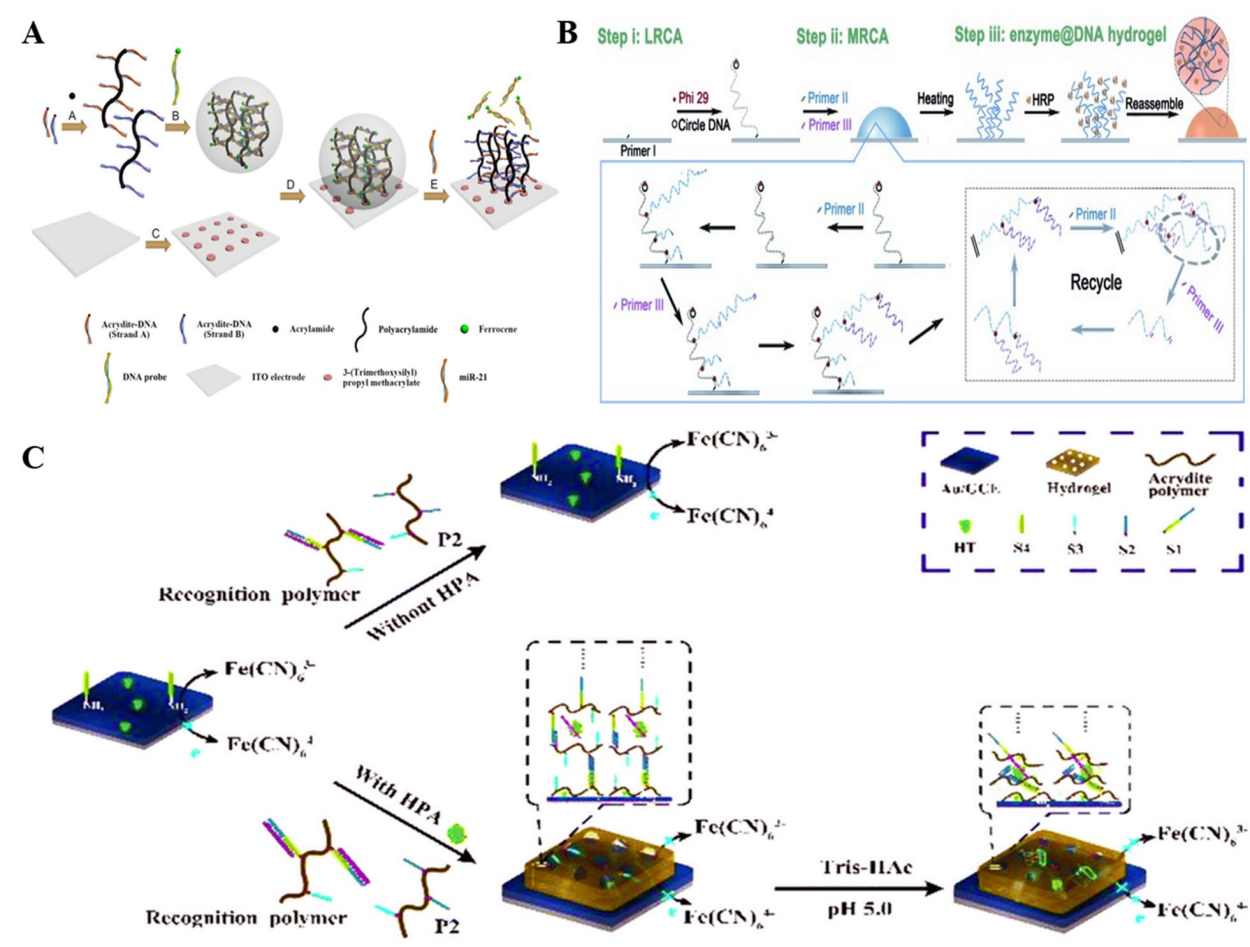
Schematic illustration of stimuli-responsive hydrogel-based electrochemical biosensing strategies. **A** The self-assembly DNA hydrogel is immobilized on ITO electrode and miR-21 can induce the dissociation of it through recognizing with probe. Reproduced with permission from Ref. [139]. **B** Surface-immobilized enzyme@DNA hydrogel formed by surficial primer-induced (SPI) strategy. Reproduced with permission from Ref. [140]. **C** The hydrogel film immobilizes on electrode through HPA and pH induces the crosslinked density change. Reproduced with permission from Ref. [142]

The electrochemical biosensor offers high sensitivity, requires low sample volume, responds quickly, and has convenient handling and portability [138]. Owing to these advantages, electrochemical biosensors based on stimulus-responsive DNA hydrogels were developed. For example, Ding’s group fabricated an electrochemical biosensor based on DNA-hybridized hydrogels for miRNA-21 detection [139]. As shown in Fig. 6A, ferrocene-labeled recognition probes are used as cross-linked strands to form a hybrid DNA hydrogel immobilized on an indium tin oxide/polyethylene terephthalate (ITO/PET) electrode. When the target miRNA-21 is added, the crosslinked chain dissociates with its recognition, the hydrogel disintegrates, the ferrocene label decreases, and the measurable current reduces. It can be effectively detected by cyclic voltammetry (CV) and differential pulse voltammetry (DPV). Results show that the method is well linear in the range of 10 nM-50 μM and has great potential as a sensitive detection platform.

The pure DNA hydrogel is easily molded into the desired size and shape by controlling the raw material concentration and reaction time. The first study of pure DNA hydrogels immobilized on an electrode surface came from Li’s group [140]. In situ growth of DNA hydrogel soft scaffolds on transparent ITO electrodes enabled simultaneous colorimetric and electrochemical measurements. As shown in Fig. 6B, primer I bound at the electrode surface can trigger linear rolling circle amplification (LRCA) to form long ssDNA, and the added primer II and primer III can bind ssDNA for multi-primed rolling circle amplification (MRCA), thus self-assembling to form pure DNA hydrogels. The hydrogel can encapsulate an extensive number of enzymes and provide a catalytic system. Due to the molecular sieving effect and good swelling/de-swelling behavior, sensitive detection of targets in serum samples can be achieved directly. In addition, the synthesized hydrogels show good long-term stability and cycling stability under different conditions. The study shows that the combination of DNA hydrogels and surface biosensing devices can broaden the scope and enhance the flexibility of DNA hydrogel applications in bioanalysis. This work will stimulate the exploration of surface immobilized DNA hydrogels for wider applications in 3D interfacial biosensing systems. In 2019, Deng et al. reported a pure DNA hydrogel structure based on non-template thermostable amplification formation, enabling ultrasensitive high-throughput detection of miRNAs on electrode microarrays [141]. After binding to the target, the exposed 3′-hydroxyl terminus of the probe DNA on the electrode surface will spread the raw material bio-dATPs by the non-template extension of TdT, and then proceed to self-multi-furcation after the addition of T_20_G_5_ sequence, forming a DNA hydrogel after continuous cycling. Avidin-HRP can be anchored to the hydrogel by streptavidin-biotin interactions and oxidized TMB as a proton donor for electroreduction, allowing detection by voltammetry and amperometry. This strategy provides a universal easy-to-perform gel generation formulation that is expected to be an alternative method for early diagnosis of disease-associated DNA, non-coding RNA and proteins (through the use of aptamers).

The hindering effect of DNA hydrogels on the electron transfer on the electrode surface affords the possibility of their use in the construction of impedance biosensors. Yang and coworkers developed a DNA hydrogel-based electrochemical impedance biosensor for the detection of heparanase (HPA) [142]. As in Fig. 6C, a cross-linked strand of DNA hydrogel is immobilized on the surface of the electrode and used as a capture probe. The two DNA elements grafted by the linear polymer are associated with HPA and pH response, respectively. When HPA is present, binding of the HPA-responsive element, in turn, induces the two DNA elements to hybridize with the capture DNA to form a DNA hydrogel membrane. As the pH drops to 5.0, the pH-responsive element can form an I-motif structure, the hydrogel density increases, and the signal is further amplified. The constructed impedance biosensor can detect HPA down to 0.003 pg mL^−1^ and has preliminary application prospects. Tang’s group assembled DNA hydrogels based on Hg^2+^-induced targeting cycle of Mg^2+^-specific DNAzyme and HCR to construct an electrochemical impedance biosensor, for sensitive and selective detection of Hg^2+^ [143]. The hairpin H1 is fixed on the electrode, and then the Hg^2+^-dependent cleavage activity of DNAzyme is used for cyclic amplification of ssDNA signal. The ssDNA can open hairpin H1, and the exposed sequence of the opened hairpin triggers HCR of H2 and H3, which are assembled layer by layer to form DNA hydrogel. The impedance sensor has a lower detection limit of 0.042 pM and a broad detection range of 0.1 pM-10 nM, which is potentially advantageous for Hg^2+^ detection in complex matrices such as river water.

While DNA hydrogel-based electrochemical sensing has excellent signal amplification capabilities, the future challenge is to develop DNA hydrogel-based electrochemical sensing strategies that are more stable and can operate in a variety of environments.

### DNA hydrogels in POCT application

With portability, stability, and ease of operation, POCT devices are particularly suitable for scenarios with limited resources, such as less industrialized countries, remote or disaster-stricken areas, and home healthcare [144–146]. And volumetric or phase change properties of DNA hydrogels in response to stimulus are considered as ideal signal transduction strategies for designing POCT devices [147, 148].

**Fig. 7:**
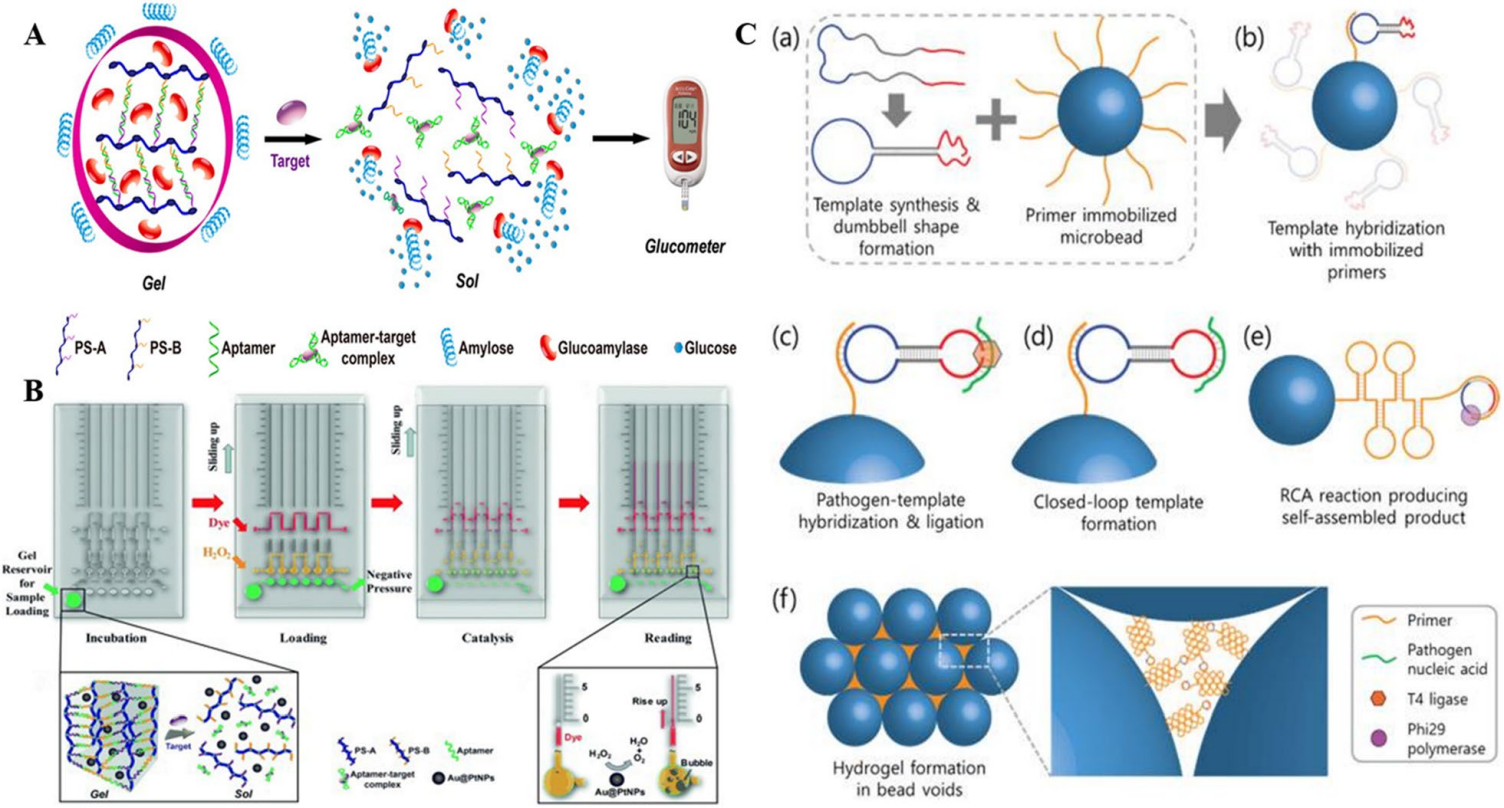
Schematic illustration of stimuli-responsive hydrogel-based POCT applications. **A** Target-responsive hydrogel PGM Readout for quantitative POCT. Reproduced with permission from Ref. [70]. **B** Integrating Au@Pt nanoparticle encapsulated DNA hydrogel with volumetric bar-chart chip for quantitative POCT. Reproduced with permission from Ref. [150]. **C** Schematic diagram of DNA gel formation through RCA in bead voids. Reproduced with permission from Ref. [158]

In this pursuit, personal glucose meters (PGMs), one of the most established commercially available quantitative portable devices, are widely used in DNA hydrogel-based biosensor manufacturing. For example, Tan and coworkers used aptamer cross-linked DNA hydrogels for signal transduction and PGMs as signal readout devices for non-glucose target quantification [70]. As shown in Fig. 7A, the DNA hydrogel formed by the aptamer cross-linking is encapsulated with glucoamylase. Upon target introduction, the aptamer recognizes and dissociates, and the disintegrating hydrogel releases the glucoamylase, which are released from the bundle into the solution to hydrolyze amylose into glucose for quantitative readout by PGM. The method only requires switching the aptamer sequence to achieve the detection of different targets. Si et al. based on the above work designed the cross-linked strands of DNA hydrogels as multicomponent nucleases (MNAzymes) to enable the detection of miRNAs [50]. Further signal amplification is achieved as the MNAzyme activated by target addition can catalyze the cleavage of multiple substrate chains. Using DNA tetrahedral hydrogels as signaling transducers, Gao et al. achieved in situ analysis of DNA methyltransferase (MTase) activity based on PGM [149]. In this work, commercially available glucose test strips are used as reaction substrates, loaded with DNA hydrogels encapsulating glycosylase and reaction substrates. Reaction readings are taken by phase change, and in contrast to the work described above, all processes for this analysis are performed on paper, eliminating the need for multiple separation and washing steps, facilitating its use in field assays. Although glucose meters can be quantitative, they still require external electronics, so an integrated strategy combining DNA hydrogels with a volumetric bar graph chip (V-chip) for “detector-free” testing has been developed [150]. As shown in Fig. 7B, DNA hydrogels encapsulating Au@PtNPs are synthesized with the aptamer as the crosslinker and stored in the repository in the lower left corner of the V-chip. Au@PtNPs can catalyze the production of oxygen from H_2_O_2_, generating pressure to drive the flow of liquid. After adding the target, the disintegrating DNA hydrogel releases Au@PtNPs, at which time the top layer of the chip is slid to form three channels in the horizontal direction, the bottom channel (green) applying negative pressure to attract the supernatant containing Au@PtNPs, the middle channel (yellow) adding H_2_O_2_ substrate, and the top channel (red) adding red ink as a color indicator. After completion, continue to slide the chip, the horizontal channel will be separated and the vertical channel will be connected. The oxygen generated after the catalytic reaction can drive the ink to move up in the tube, and the moving distance in a certain time is proportional to the concentration of the target, thus achieving visual quantitative detection. Adopting the similar approach, this group introduced an immunoaffinity column (IAC) portable enrichment technique to achieve the detection of Ochratoxin A (OTA) in beer, obtaining high sensitivity and low detection limit (0.51 ppb), which is beneficial to ensure food safety and human health [151]. The same group successfully achieved quantitative detection of Pb^2+^ using V-chip by designing the cross-linking agent as a Pb^2+^-dependent DNAzyme [152]. However, due to some limitations in fabrication and pretreatment of the V-chip, the group went on to combine a Pb^2+^-dependent DNAzyme hydrogel with a handheld pressuremeter for quantitative POCT and obtained sensitive detection results with simple sample processing [153]. The method could also be used in combination with an electronic balance for the detection of aflatoxin B1(AFB1) [69].

The DNA hydrogel-based microfluidic system provides inspiration for the development of a reliable and affordable POCT platform. Compared to surface-based nucleic acid systems, hydrogels have advantages in terms of solution-like environment and non-contaminating properties, especially in applications with complex matrices [154]. In addition, the stability of hydrogels allows for normal reaction under demanding mixing or flow conditions [155].Utilizing a microfluidic system, Lee et al. constructed an in-situ formed DNA hydrogel using isothermal amplification to detect the pathogens in viruses or bacteria, and the system was named as DhiTACT [156]. As the solution to be tested flows through the microfluidic channel, the ssDNA of the pathogen can bind to the target DNA to excite the isothermal amplification to form long ssDNA. Subsequently, DhiTACT allows the product to self-assemble to form a DNA hydrogel, which in turn blocks the microfluidic channel, thus, enabling the detection of Bacillus anthracis and Ebola virus. However, the sensitivity of the developed system is restricted by a large number of the DNA strands required for the gel to block the microfluidic channel. Subsequently, authors developed an improved system, named as DhITACT-TR (TRail) [157]. The next-generation system polymerizes the surface-associated isothermal RCA of the target nucleic acid coupled with TRail for the rapid and sensitive detection of the Middle East Respiratory Syndrome (MERS) virus in the pseudo-serum samples. With fluorescence optical detection, a rapid diagnosis of the MERS virus can be achieved within 30 min with a 100-fold improvement in sensitivity. Compared with the current diagnostic platforms, TRail can provide a superior cost-effectiveness. With high potential for clinical application, it is expected to be an outstanding diagnostic platform for the prevention of the infectious diseases. Similarly, a new microfluidic device has been developed that grows the DNA hydrogels on the surface of numerous microbeads filled in the microfluidic channels, thus, blocking the flow paths formed between the microbeads [158]. As shown in Fig. 7C, the RCA process takes place on the surface of each microbead, and the increased surface area for reaction and reduced cross-sectional area of the channels lead to a significant reduction in the detection time (less than 15 min). Subsequently, the detection limit is enhanced by 10-100 folds as compared to the other detection systems. By integrating a multi-channel design, multiple analyses can be performed simultaneously, and the system can be practically used for screening in airports and infectious disease transmission areas.

In recent years, the applications of DNA hydrogels in microfluidic paper-based analytical devices (μPADs) have also been extensively investigated, where paper is readily available, stable, and can also be folded to form multilayer microfluidic channels [159, 160]. In 2015, Wei and colleagues combined DNA hydrogels with μPADs to build a versatile POCT platform [161]. The aptamer is used as a cross-linking agent for the DNA hydrogel, and the stable hydrogel prevents the flow of colored dye in the absence of the target. In the presence of the target, the DNA hydrogel disintegrates and the colored liquid flowing down can be observed in the detection area. The method enables simultaneous semi-quantitative detection of adenosine, cocaine and Pb^2+^. Subsequently, the same research group introduced a new approach that implemented different forms of output. The specific principle is to use DNA hydrogels to encapsulate amylase, and the amylase released from the disintegrated hydrogels catalyzes the hydrolysis of amylose to glucose, then the resulting glucose is oxidized by GOx to generate H_2_O_2_, which acts with HRP on different substrates to achieve different signal outputs. When catalyzed with colorless 3,3′-diaminobenzidine (DAB), brown insoluble poly-3,3′-diaminobenzidine [Poly (DAB)] is produced, relying on μPAD for visual distance quantitative readings [162]. When applied to colorless iodide, the iodine generated is colored in the detection zone of μPAD, allowing not only semi-quantitative detection by the naked eye, but also the capture of images by electronic devices, which can be analyzed and then saved and transported via cloud computing for telemedicine [163].

The current DNA-based hydrogels are relatively expensive to prepare, hindering their practical application as disposable devices in POCT scenarios. In the future, reducing the cost of DNA hydrogel synthesis or improving the reusability of DNA hydrogels becomes the direction of effort.

### DNA hydrogels in other biosensing signals

#### DNA hydrogel-based low-field nuclear magnetic resonance (LF-NMR) biosensing

**Fig. 8:**
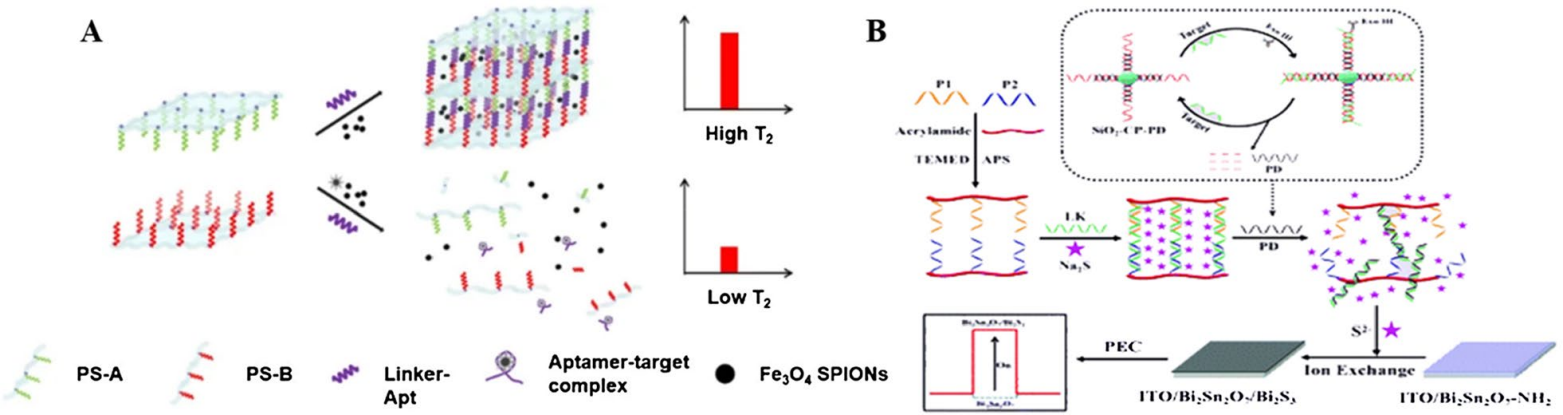
Schematic illustration of stimuli-responsive hydrogel-based other biosensing applications. **A** Schematic of the LF-NMR biosensor based on Switchable DNA hydrogel. Reproduced with permission from Ref. [86]. **B** Schematic representation for the PEC biosensor based on PD-responsive DNA hydrogel and Bi_2_Sn_2_O_7_/Bi_2_S_3_heterojunction. Reproduced with permission from Ref.[51]

Recently, Wang and colleagues reported a nanoprobe based on LF-NMR and stimuli-responsive DNA hydrogels for sensitive and efficient detection of bisphenol A (BPA) [86]. As shown in Fig. 8A, the three-dimensional polymer network of the DNA hydrogel allows for the sustainable release of the encapsulants, thereby simplifying the experimental manipulation of LF-NMR and reducing sample loss. In this work, DNA hydrogels encapsulating Fe_3_O_4_ superparamagnetic iron oxide nanoparticles (SPIONs) are synthesized using the target aptamer as a cross-linking agent, at which time the Fe_3_O_4_ SPIONs are in the aggregated state. Upon addition of the target, the aptamer dissociates by binding to it, causing the DNA hydrogel to disintegrate and the released aggregated particles to disperse, resulting in a decrease in relaxation time T2, leading to quantitative detection of the signal change detected by LF-NMR. Through the flexible design of the probe, the method can provide alternative assays for clinical diagnostics, environmental monitoring and food safety testing etc.

#### DNA hydrogel-based photoelectrochemical (PEC) biosensing

The PEC biosensor combines the advantages of bioanalysis and electrochemical analysis making it a promising analytical method, while the key to constructing PEC biosensors roots in the design and preparation of excellent photoactive nanomaterials. In 2021, Jie’s group found that the Bi_2_Sn_2_O_7_/Bi_2_S_3_ heterojunction formed by in situ anion exchange between S^2−^ and Bi_2_Sn_2_O_7_ sensitized the PEC signal of the photoactive material [51]. Based on this, highly sensitive detection of P53 gene was achieved using switchable DNA hydrogels and Bi_2_Sn_2_O_7_/Bi_2_S_3_ heterojunctions. As shown in Fig. 8B, a large number of product strands (PD) are generated in the target-induced DNA exonuclease (Exo III) catalytic cycle, and DNA strands complementary to PD are used as cross-linking agents to synthesize Na_2_S-coated DNA hydrogels. After adding PD to the DNA hydrogel, the cross-linked chain dissociates by binding to PD and the DNA hydrogel collapses to release Na_2_S. The released Na_2_S is introduced into the Bi_2_Sn_2_O_7_ electrode to obtain the Bi_2_Sn_2_O_7_/Bi_2_S_3_ heterojunction in situ, which enhances the photocurrent of target detection by a factor of 63. The developed PEC sensor is simple to operate and provides a promising form of sensing that can be used for the detection of different targets.

## Conclusion and perspectives

In conclusion, this study comprehensively reviews the specific applications of the stimulus-responsive DNA hydrogels in biosensing, along with exploring the recent advances. Though this field has developed over the years, however, there are several important challenges that need to be addressed so as to move forward. First, in the detection of large molecules, the slow mass transfer and slow response of the hydrogels limit their rapid detection ability, which is expected to be solved by the development of intelligent DNA hydrogels that respond more efficiently to large molecules. Second, the integration of the DNA hydrogels with various interfacial analysis systems is still challenging. It indicates that these systems are still in the minority in the field of bioanalysis. Thus, the integration of the hydrogels with the solid-phase interfaces needs to be effectively addressed. Next, the most existing biosensing systems based on the stimuli-responsive DNA hydrogels require bulky hydrogel systems, resulting in the hydrogels that require high concentrations of the target molecules to produce the phase transitions. It enhances the cost of synthesis and reduces the sensitivity of the reaction. Therefore, the development of microgels or DNA hydrogel films is one of the ways to overcome this issue in the future. In addition, due to the diversity of envisioned DNA hydrogel application scenarios, the environmental stability of DNA hydrogels needs to be improved. The presence of complex environmental matrices also places demand on the specificity of DNA hydrogels, which relies on the continuous improvement and development of aptamer screening techniques. Then, the colorimetric-based sensing methods are straightforward and have a wide prospect in POCT detection. However, the issues with sensitivity and quantification compromise their application. These can be overcome by combination with the efficient signal amplification strategies. Next, the stimuli-responsive DNA hydrogels are currently in their infancy for building the intelligent materials with built-in logic decisions. It is envisioned that the molecular logic gates constructed from the stimulus-responsive DNA hydrogels hold a significant promise for improving the information processing. Finally, it should be noted that the prevailing challenges may be gradually resolved through continuous advances in biochemistry and materials chemistry. It is expected that the fast-response, low-cost and ultra-sensitive smart DNA hydrogel biosensors will soon find uses in POCT detection, wearable and implantable biosensing devices, soft robotics, etc.

## Data Availability

Not applicable.

## Abbreviations

POCT: Point-of-care testing
YMA: Y-monomer A
YMB: Y-monomer B
GSH: Glutathione
dsDNA: Double-stranded DNA
HCR: Hybridization chain reaction
RCA: Rolling circle amplification
MCA: Multi-primed chain amplification
ssDNA: Single-stranded DNA
HRP: Horseradish peroxidase
IFSs: I-motif-sequence forming sequences
pNIPAM: Poly-N-isopropylacrylamide
QD: Quantum dot
CNT: Carbon nanotube
MOF: Metal organic framework
CD: Carbon dot
PpIX: Protoporphyrin IX
SWCNT: Single-walled carbon nanotube
CB[8]: Cucurbit[8]uril
FRET: Fluorescence resonance energy transfer
dNTP: Deoxynucleotide triphosphate
TdT: Terminal deoxynucleotidyl transferase
dATP: Deoxyadenosine triphosphate
DNase I: DNA endonuclease I
BHQ: Black hole quencher
AuNPs: Gold nanoparticles
CTAB: Cetyltrimethylammonium bromide
MC-LR: Microcystin-LR
TMB: 3,3,5,5-Tetramethylbenzidine
ABTS: 2,2′-Azino-bis (3-ethyl-benzothiozoline-6-sulfonic acid)
CRISPR: Clustered regularly interspaced short palindromic repeats
SERS: Surface-enhanced Raman spectroscopy
AFP: α-Fetoprotein
TB: Toluidine blue
DSN: Double-stranded nuclease
3-MPBA: 3-Mercaptophenylboronic acid
3-HTP: 3-Hydroxyphenol
SA: Streptavidin
SPR: Surface plasmon resonance
CL: Chemiluminescence
ECL: Electrochemiluminescent
PTC-DEDA: Amphiphilic pyrene derivative
ITO/PET: Indium tin oxide/polyethylene terephthalate
CV: Cyclic voltammetry
DPV: Differential pulse voltammetry
LRCA: Linear rolling circle amplification
MRCA: Multi-primed rolling circle amplification
HPA: Heparanase
PGM: Personal glucose meter
MNAzymes: Multicomponent nucleases
MTase: Methyltransferase
V-chip: Volumetric bar graph chip
IAC: Immunoaffinity column
OTA: Ochratoxin A
AFB1: Aflatoxin B1
MERS: Middle East Respiratory Syndrome
μPADs: Microfluidic paper-based analytical devices
DAB: 3,3′-Diaminobenzidine
LF-NMR: Low-field nuclear magnetic resonance
BPA: Bisphenol A
SPIONs: Superparamagnetic iron oxide nanoparticles
PEC: Photoelectrochemical
PD: Product strand
